# Molecular Subtypes and Biomarkers of Ulcerative Colitis Revealed by Sphingolipid Metabolism-Related Genes: Insights from Machine Learning and Molecular Dynamics

**DOI:** 10.3390/cimb47080616

**Published:** 2025-08-04

**Authors:** Quanwei Li, Junchen Li, Shuyuan Liu, Yunshu Zhang, Jifeng Liu, Xing Wan, Guogang Liang

**Affiliations:** 1 Department of General Surgery, The First Affiliated Hospital of Dalian Medical University, Dalian 116000, China; liqw@dmu.edu.cn (Q.L.); lijc03@dmu.edu.cn (J.L.); liusy22@dmu.edu.cn (S.L.); zhangys01@dmu.edu.cn (Y.Z.); liujf01@dmu.edu.cn (J.L.); 2 Institute of Integrative Medicine, Dalian Medical University, Dalian 116000, China

**Keywords:** ulcerative colitis, sphingolipid metabolism, machine learning, biomarkers, molecular subtypes, molecular docking, molecular dynamics simulations

## Abstract

Ulcerative colitis (UC) is a chronic inflammatory bowel disease associated with disrupted lipid metabolism. This study aimed to uncover novel molecular subtypes and biomarkers by integrating sphingolipid metabolism-related genes (SMGs) with machine learning approaches. Using data from the GEO and GeneCards databases, 29 UC-related SMGs were identified. Consensus clustering was employed to define distinct molecular subtypes of UC, and a diagnostic model was developed through various machine learning algorithms. Further analyses—including functional enrichment, transcription factor prediction, single-cell localization, potential drug screening, molecular docking, and molecular dynamics simulations—were conducted to investigate the underlying mechanisms and therapeutic prospects of the identified genes in UC. The analysis revealed two molecular subtypes of UC: C1 (metabolically dysregulated) and C2 (immune-enriched). A diagnostic model based on three key genes demonstrated high accuracy in both the training and validation cohorts. Moreover, the transcription factor FOXA2 was predicted to regulate the expression of all three genes simultaneously. Notably, mebendazole and NVP-TAE226 emerged as promising therapeutic agents for UC. In conclusion, SMGs are integral to UC molecular subtyping and immune microenvironment modulation, presenting a novel framework for precision diagnosis and targeted treatment of UC.

## 1. Introduction

Ulcerative colitis (UC) is a chronic, recurrent, and nonspecific inflammatory disorder primarily affecting the colon’s mucosa and submucosa. In recent years, both the incidence and prevalence of UC have steadily risen [1]. The etiology of UC remains unclear and multifactorial; however, increasing evidence suggests that, in addition to genetic factors, disturbances in the intestinal flora, the host immune system, environmental influences, abnormal lipid metabolism, and inflammation play a significant role [2,3]. Despite advancements in treatment, including drug and endoscopic therapies, clinical outcomes remain suboptimal [4]. The disease’s heterogeneity and its complex molecular and immunological mechanisms highlight the urgent need for novel biomarkers and therapeutic targets.

Sphingolipids, a type of bioactive lipid, are crucial for maintaining the integrity and function of cell membranes [5]. These molecules are composed of a polar head group, a fatty-acid chain, and a sphingosine backbone. They are involved in various biological processes, including signal transduction, lipid raft formation, and cell adhesion [6]. Previous studies have indicated that certain sphingolipid metabolites and the sphingosine 1-phosphate signaling pathway may play a role in regulating UC-related inflammation [7]. Additionally, sphingosine kinase 1—which can protect against intestinal injury and systemic inflammation through genetic knockout or pharmacological inhibition—has been identified as a key regulator in dextran sulfate sodium-induced colitis [8]. Therefore, investigating the expression and function of sphingolipid-related genes in UC is critical for advancing both our understanding of the disease and the development of therapeutic strategies.

In research related to disease states, machine learning has emerged as a powerful tool for analyzing complex biological data. Techniques such as Random Forest (RF), Support Vector Machine (SVM), and LASSO regression can integrate and analyze large datasets to uncover patterns and relationships that traditional methods may overlook [9,10]. This study aims to employ machine learning to identify sphingolipid metabolism-related genes (SMGs) associated with UC, and to delineate sphingolipid metabolism-driven molecular subtypes and biomarkers. The goal is to enhance early diagnosis and improve treatment strategies for UC. 

## 2. Methods

**Figure 1:**
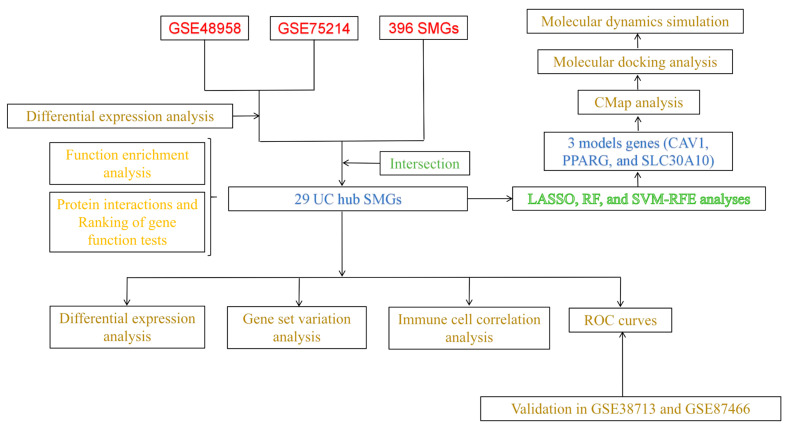
Flow chart of this study.

The study design flowchart is presented in Figure 1.

### 2.1. Raw Data Sources

From the GeneCards database (https://www.genecards.org/; accessed on 3 May 2025), 396 SMGs (relevance score > 10) were identified [11]. Gene expression datasets related to UC were retrieved from the GEO database (accessed on 3 May 2025), including GSE48958 [12], GSE75214 [13], GSE38713 [14], and GSE87466 [15]. These datasets were divided into a training group (GSE48958 and GSE75214) and validation sets (GSE38713 and GSE87466) to develop a robust and reliable UC prediction model. Supplementary Table S1 lists information on clinical characteristics of samples from the four datasets.

### 2.2. Integrated Analysis of UC-Related SMGs

To eliminate batch effects between arrays, the “sva” (version 3.42.0) R package was employed on the combined training matrix [16]. Differentially expressed genes (DEGs) between the UC and control groups were determined on the integrated dataset, with criteria set to an adjusted *p*-value < 0.05 and |log2FC| > 1. Statistical significance was assessed using moderated *t*-tests implemented in the “limma” (version 3.50.3) package, with multiple testing correction performed by the Benjamini–Hochberg method to control the false discovery rate (FDR). UC-related SMGs were identified by intersecting the SMGs with the DEGs using a Venn diagram.

### 2.3. Functional Enrichment Analysis

The UC-related DEGs were uploaded to the STRING database (accessed on 10 May 2025) to explore protein–protein interaction (PPI) networks [17]. GeneMANIA (https://genemania.org/) (accessed on 10 May 2025) was used to rank genes based on functional testing [18]. Functional enrichment analysis of these DEGs was conducted through the Metascape database (accessed on 10 May 2025) [19]. Enrichment significance was assessed using the cumulative hypergeometric distribution, and *p*-values were adjusted for multiple comparisons using the Benjamini–Hochberg FDR correction. Enriched terms were filtered based on the criteria of *p* < 0.01, a minimum gene count ≥ 3, and an enrichment factor > 1.5.

### 2.4. Identification of Sphingolipid Metabolism-Related Molecular Subtypes in Patients with UC

To classify UC samples based on the expression levels of UC-related SMGs, the “ConsensusClusterPlus” (version 1.58.0) R package was applied [20]. Principal component analysis (PCA) was performed to validate the clustering results, followed by biological characteristic comparisons of the different subgroups. To assess the differences in immune cell infiltration levels between UC molecular subtypes, Wilcoxon’s rank-sum test was applied to each immune cell type. Statistical significance was determined using two-sided tests, and *p*-values were adjusted for multiple comparisons.

### 2.5. Identifying the Best Model Genes of UC-Related SMGs

To prevent overfitting, LASSO analysis was performed using the “glmnet” (version 4.1.8) R package, with 10-fold cross-validation used to determine the optimal penalty parameter λ [21,22]. RF analysis was conducted using the “randomForest” (version 4.7.1.1) R package (ntree = 500) [23]. The mean decrease in Gini index produced by the RF was used to assess feature importance, with genes having relative importance greater than one classified as characteristic genes. Additionally, SVM-RFE was implemented using the “e1071” (version 1.7.14) R package [24]. SVM-RFE, based on structural risk minimization, aims to minimize empirical error and maximize learning performance. The model genes selected for further analysis were those that intersected across the three methods. To compare the expression levels of model genes between control and UC groups, Wilcoxon’s rank-sum test was employed. Significance was determined using two-sided tests, and *p*-values were annotated as significance levels in the boxplots.

### 2.6. Gene Set Variation Analysis (GSVA) and Immunoinfiltration Analysis

To investigate pathway activity alterations in the model genes, GSVA was performed using the “GSVA” (version 1.42.0) R package [25]. For each pathway, a two-sided unpaired Student’s *t*-test was used to assess the difference in ssGSEA scores between the two groups. Pathways were considered significantly upregulated or downregulated based on the *p*-value (<0.05) and the direction of the t-statistic. Simultaneously, a comprehensive evaluation of immune cell composition—an essential aspect of UC research—was conducted using the CIBERSORT algorithm [26]. This efficient method facilitated the quantification of 22 distinct immune cell types. The correlations between gene expression levels and immune cell infiltration were assessed using Spearman’s rank correlation coefficient. The analysis was visualized with lollipop plots, where the circle size denotes the magnitude of the correlation, and the color represents the statistical significance of the association. A *p*-value threshold of 0.05 was used to define statistically significant correlations.

### 2.7. Cell Culture and Quantitative Real-Time PCR Analysis

The Caco-2 cell line was cultured in MEM supplemented with 20% fetal bovine serum and 1% penicillin–streptomycin. Consistent with prior research, the cells were treated with LPS (1 µg/mL) for 24 h to induce a colitis model [27,28]. Total RNA was extracted using Trizol reagent, and cDNA synthesis was performed using a reverse-transcription kit. Gene expression levels were quantified via a fluorescent dye-based assay with SYBRGreen I. The expression levels of target genes between the two groups were compared using a *t*-test, while RNA expression was evaluated and quantified through the ΔΔCt method. A *p*-value < 0.05 was considered statistically significant.

### 2.8. Prediction of Core Gene Transcription Factors (TFs), Cell Localization, and Single-Cell Profiling Analysis

The model genes for TFs were identified using the TFTF online tool (accessed on 15 May 2025) [29], which integrates three major TF-target databases: JASPAR [30], GTRD [31], and ChIP_Atlas [32]. To explore the localization patterns of key genes in the colon, single-cell transcriptome data were obtained from the Human Protein Atlas (HPA) database (accessed on 15 May 2025).

### 2.9. Prediction of Potential Therapeutic Drugs for UC

The Connection Map (CMap, https://clue.io/) database (accessed on 15 May 2025) was used to explore functional linkages between genes, small-molecule drugs, and diseases [33,34]. UC-related DEGs were uploaded to the CMap database to predict potential therapeutic drugs. The identified small-molecule compounds were subsequently validated through molecular docking. The 3D structures of target proteins were retrieved from the Protein Data Bank (PDB), and docking was performed using the CB-Dock2 online server (https://cadd.labshare.cn/cb-dock2/index.php), which combines cavity detection with AutoDock Vina to automatically identify optimal binding pockets and docking poses (accessed on 18 May 2025). Molecular dynamics simulations were conducted using GROMACS 2021.3 with the CHARMM36 force field.

## 3. Results

### 3.1. Identification of DEGs Associated with UC and SMGs

**Figure 2:**
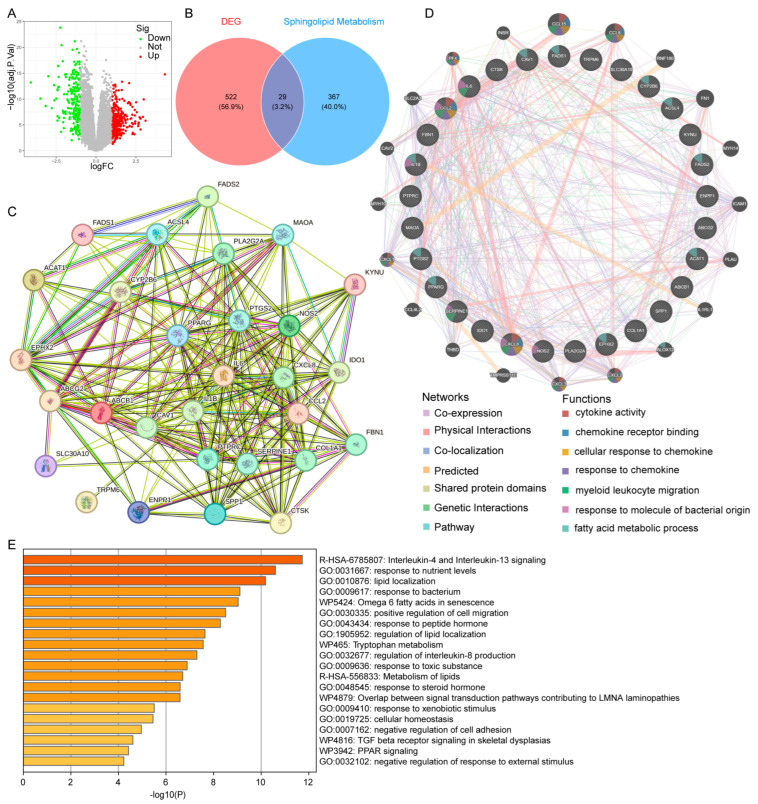
Identifying UC-related SMGs: (**A**) volcano plot of gene expression differences between UC and control groups; (**B**) Venn diagram showing the intersection of SMGs and DEGs from UC; (**C**) PPI network illustrating interactions of the 29 intersecting genes; (**D**) GeneMANIA analysis of UC-related SMGs; (**E**) functional enrichment analysis of the UC-related SMGs.

Raw UC data were sourced from the GEO database, and after removing batch effects, the datasets were normalized (Figure S1A–D). “Limma” analysis was conducted on the UC cohort, identifying 551 DEGs, including 340 upregulated and 211 downregulated genes (Figure 2A). Further intersection of UC-related DEGs with SMGs revealed 29 shared genes for subsequent analysis (Figure 2B). The PPI network for these 29 DEGs was constructed using data from the STRING and GeneMANIA databases (Figure 2C,D). Functional enrichment analysis indicated that these genes were predominantly involved in interleukin-4 and interleukin-13 signaling, nutrient response, and lipid localization (Figure 2E).

### 3.2. Identification of SMG-Related Clusters in UC

**Figure 3:**
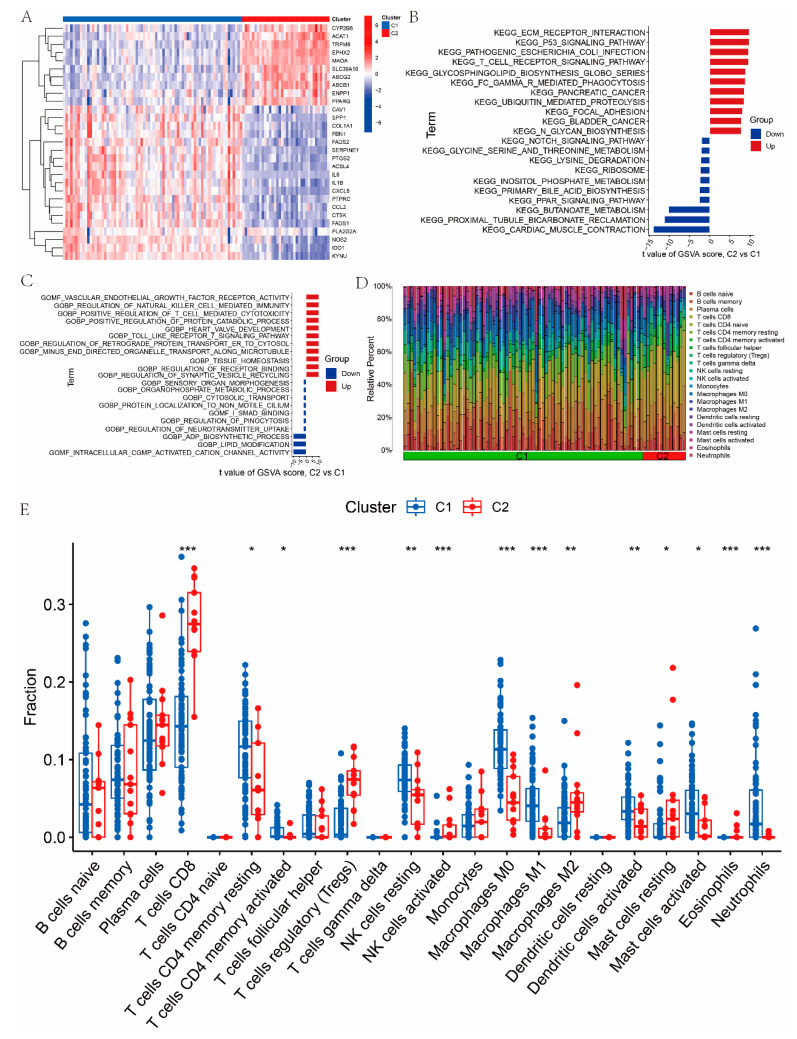
Identification and characterization of two SMG-related molecular subtypes in UC: (**A**) heatmap showing the expression profiles of UC-SMGs across the two identified clusters (C1 and C2); (**B**) GSVA results showing differences in functional enrichment between C1 and C2, based on KEGG pathways; (**C**) GSVA results showing differences in functional enrichment between C1 and C2, based on GO gene sets; (**D**,**E**) immune cell infiltration patterns in C1 and C2 subtypes assessed by CIBERSORT, indicating subtype-specific immune characteristics. ( * *p* < 0.05, ** *p* < 0.01, and *** *p* < 0.001).

To classify UC samples, a consensus clustering approach was applied based on the expression patterns of the 29 UC-SMGs. The results were most stable when the samples were divided into two clusters (Figure S1E,F). The expression levels of the 29 UC-related SMGs were then compared between the two clusters, C1 and C2, to assess molecular differences (Figure 3A). Next, GSVA was performed to identify potential biological and functional distinctions between the clusters. The results showed that C1 (the metabolically dysregulated subtype) was mainly associated with butanoate metabolism, glycine–serine metabolism, and lipid modification, whereas C2 (the immune-enriched subtype) was enriched in the positive regulation of T-cell-mediated cytotoxicity, the regulation of natural killer cell-mediated immunity, and the T-cell receptor signaling pathway (Figure 3B,C). Further comparison of immune cell infiltration between the two clusters revealed that T cells (CD4 memory), resting NK cells, macrophages (M0 and M1), activated dendritic cells, and neutrophils were more abundant in C1. In contrast, T cells (CD8), regulatory T cells (Tregs), and M2 macrophages were significantly more abundant in C2 (Figure 3D,E).

### 3.3. Construction of the Diagnostic Model for UC

**Figure 4:**
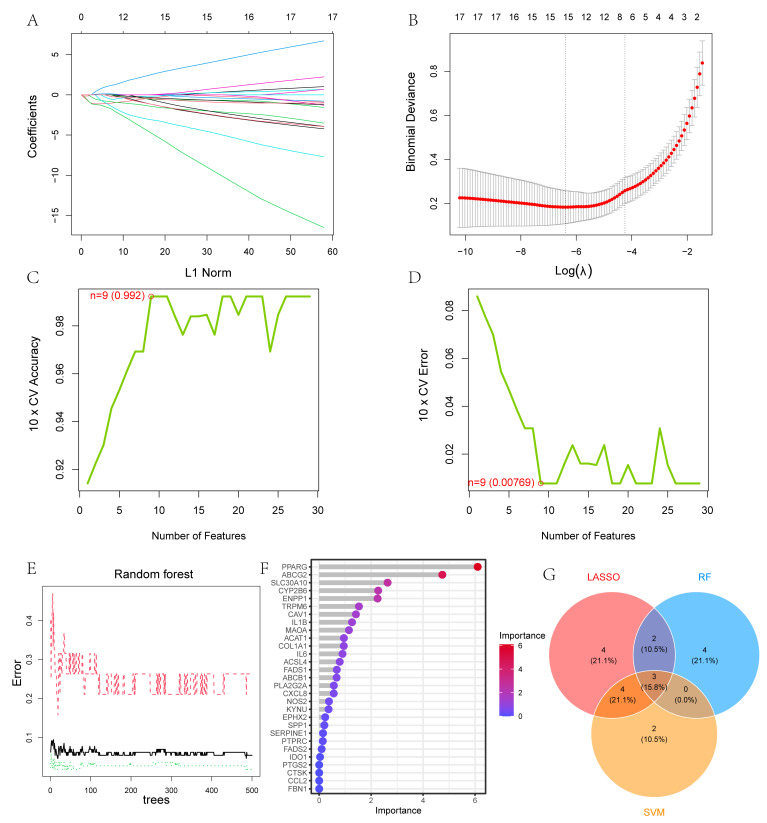
Use of machine learning technology to construct a UC diagnostic model: (**A**,**B**) thirteen genes selected by LASSO regression. Different colors correspond to different variables; (**C**,**D**) nine genes identified using SVM-RFE; (**E**,**F**) six genes with importance ratings greater than 1 identified using RF. The black solid line shows the overall out-of-bag (OOB) error rate. The red dashed and green dotted lines represent the class-specific OOB error rates for the Control and UC groups, respectively; (**G**) Venn diagram displaying shared genes across the three machine learning models.

Three machine learning models were developed based on the 29 UC-related SMGs. The LASSO regression approach identified 13 genes as potential diagnostic indicators (Figure 4A,B). Using the SVM-RFE method, nine genes were further selected as potential biomarkers from this set (Figure 4C,D). Additionally, nine genes with importance values greater than one, as determined by the RF algorithm, were included for further investigation (Figure 4E,F). By overlaying the results from all three methods on a Venn diagram, three genes—CAV1, PPARG, and SLC30A10—were identified as key diagnostic biomarkers (Figure 4G).

### 3.4. Evaluation of the Diagnostic Model

**Figure 5:**
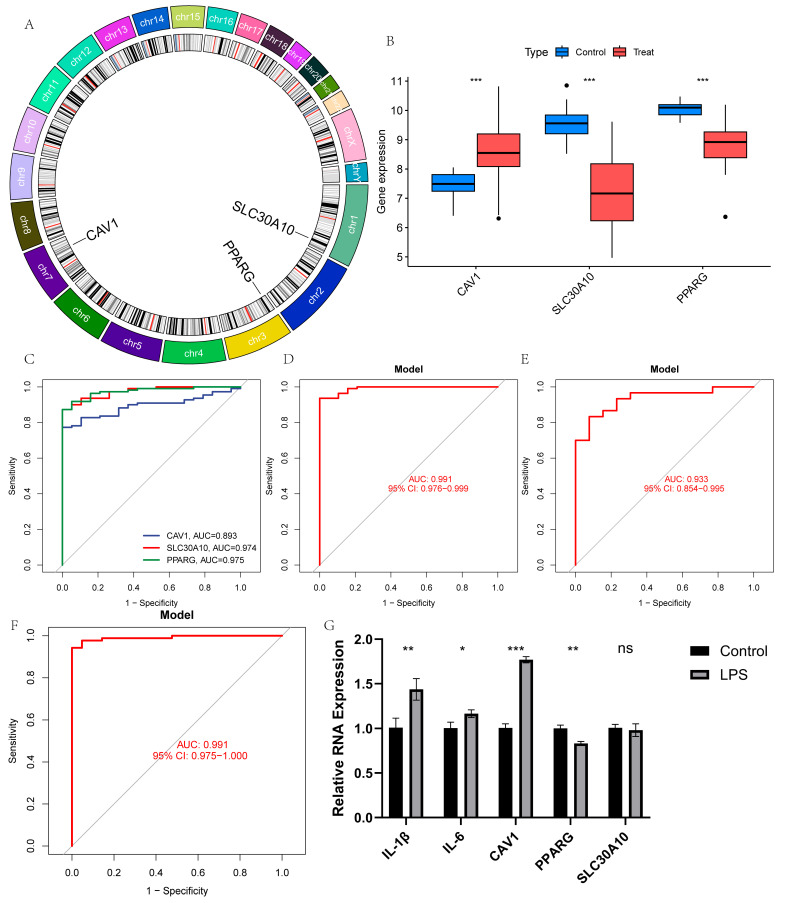
Diagnostic effect of the three-gene model on UC: (**A**) chromosomal locations of the three model genes; (**B**) box plots showing expression differences in CAV1, PPARG, and SLC30A10 between UC and normal samples in the training set; (**C**,**D**) ROC curves for the three genes and the model in the training set; (**E**,**F**) ROC curves for the three genes and the model in the validation set; (**G**) expression levels of inflammatory factors and the three model genes in the cell model. (ns, non-significant, * *p* < 0.05, ** *p* < 0.01, and *** *p* < 0.001).

The chromosomal locations of the three model genes are shown in Figure 5A. Among them, CAV1 was highly expressed in patients with UC, while PPARG and SLC30A10 were downregulated (Figure 5B). These findings were confirmed in the validation set (Figure S2A,B). The receiver operating characteristic (ROC) curves indicated strong predictive values for all three genes (Figure 5C). Notably, the three-gene prediction model, with an AUC of 0.991, exhibited superior performance (Figure 5D). To further assess the model’s accuracy, the same analysis was conducted on the validation set, demonstrating high predictive value in this cohort as well (Figure 5E,F). Additionally, UC cellular models were generated by treating Caco-2 cell lines with LPS, and the expression levels of the three model genes were confirmed. The UC model was successfully established, as shown by significantly elevated levels of IL-6 and IL-1β in the model group compared to the control group (Figure 5G). The expression levels of the three model genes in the cellular model closely aligned with the bioinformatics findings (Figure 5G).

### 3.5. Analysis of the Functional Enrichment and Immune Infiltration of the Model Genes

**Figure 6:**
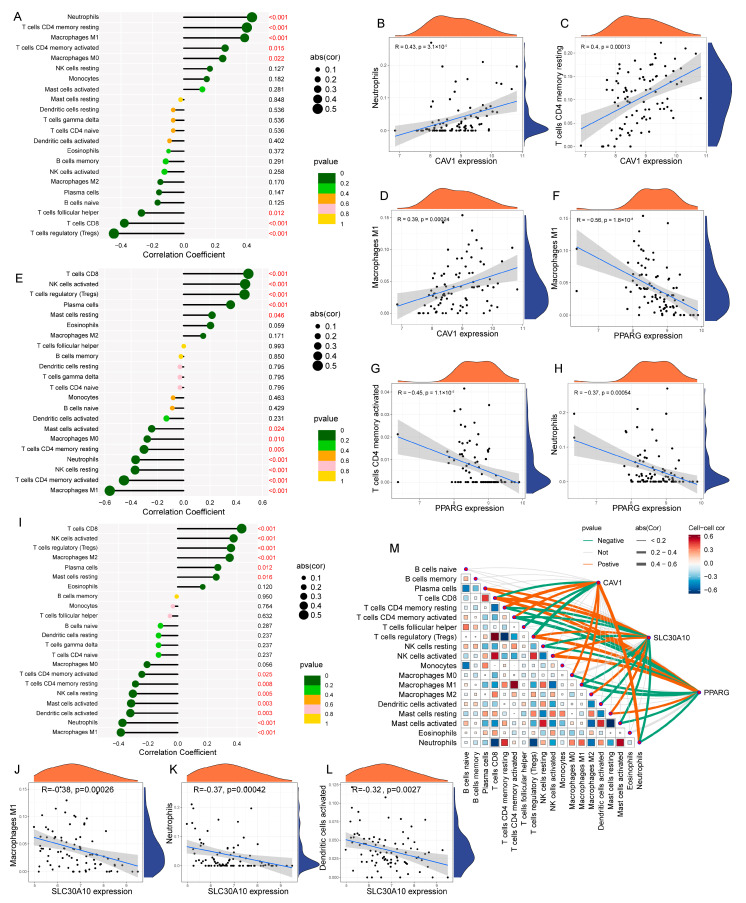
Correlation analysis between model gene expression and immune cell infiltration in UC: (**A**) lollipop plot showing the correlations between CAV1 expression and various immune cell types; (**B**–**D**) Individual correlation plots between CAV1 and neutrophils, resting CD4 memory T cells, and M1 macrophages, respectively; (**E**) lollipop plot showing correlations between PPARG and various immune cells; (**F**–**H**) detailed correlations of PPARG with M1 macrophages, activated CD4 memory T cells, and neutrophils; (**I**) lollipop plot showing correlations between SLC30A10 and various immune cells; (**J**–**L**) detailed correlations of SLC30A10 with M1 macrophages, neutrophils, and activated dendritic cells; (**M**) summary plot showing the correlations between the three model genes and immune cell subsets, along with inter-cellular correlations.

To explore the pathway enrichment differences in the three model genes, GSVA analysis was performed. CAV1 was found to be highly expressed in lipid metabolism pathways, including the diacylglycerol metabolic process, pyruvate metabolism, and inositol phosphate metabolism. In contrast, PPARG and SLC30A10 were downregulated in processes such as monoacylglycerol metabolism, retinol metabolism, nitrogen metabolism, and glycerophospholipid metabolism (Figure S3). Additionally, this study explored the relationship between each model gene and various immune cell types, revealing several interesting correlations. Notably, CAV1 was positively correlated with neutrophils, resting CD4 memory T cells, and M1 macrophages, while PPARG exhibited a negative correlation with M1 macrophages, neutrophils, and activated CD4 memory T cells (Figure 6A–H). Furthermore, SLC30A10 showed a negative correlation with activated dendritic cells, neutrophils, and M1 macrophages (Figure 6I–L). This study also performed a correlation analysis between the different immune cell types in patients with UC and examined gene–immune cell interactions (Figure 6M).

### 3.6. Screening of Transcription Factor (TFs) of Model Genes and Single-Cell Expression Analysis

TFs are proteins that regulate transcription by binding to DNA in a sequence-specific manner, ultimately controlling the expression of target genes and influencing biological phenotypes and pathological processes. To identify TFs associated with CAV1, PPARG, and SLC30A10, three datasets were merged (Figure S4A–C). The intersection of these datasets revealed a common TF, FOXA2, which could potentially regulate all three model genes (Figure S4D). Single-cell expression analysis from the HPA database showed distinct expression patterns for the three key genes in the colon. CAV1 was predominantly expressed in undifferentiated cells (Figure S4E), while PPARG and SLC30A10 were mainly localized in distal enterocytes (Figure S4F,G).

### 3.7. Screening of Potential Therapeutic Agents

**Figure 7:**
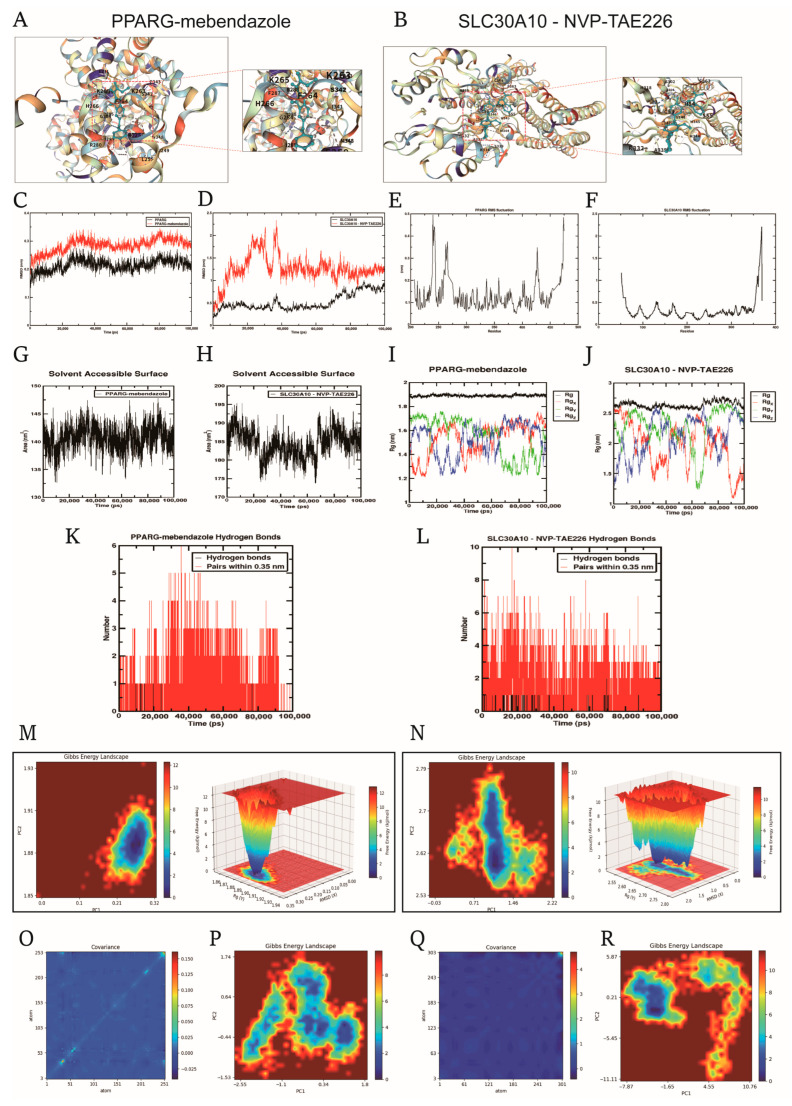
Molecular docking and molecular dynamics (MD) simulation analyses of PPARG–mebendazole and SLC30A10–NVP-TAE226 complexes to assess binding stability and interaction dynamics. Molecular docking models showing the binding poses of mebendazole with PPARG (**A**) and NVP-TAE226 with SLC30A10 (**B**); blue dashed lines indicate predicted hydrogen bonds at the active site. Root mean square deviation (RMSD) plots of the two protein–ligand complexes during 100 ns MD simulation, reflecting the overall structural stability of PPARG–mebendazole (**C**) and SLC30A10–NVP-TAE226 (**D**) systems. Root mean square fluctuation (RMSF) of protein residues in the PPARG–mebendazole (**E**) and SLC30A10–NVP-TAE226 (**F**) complexes, reflecting local flexibility upon ligand binding. Solvent-accessible surface area (SASA) of the PPARG–mebendazole (**G**) and SLC30A10–NVP-TAE226 (**H**) complexes during the simulation, reflecting potential conformational rearrangements. Radius of gyration (Rg) curves for the PPARG–mebendazole (**I**) and SLC30A10–NVP-TAE226 (**J**) complexes, assessing structural compactness. Number of hydrogen bonds formed between ligands and proteins in the PPARG–mebendazole (**K**) and SLC30A10–NVP-TAE226 (**L**) systems over time. Two- and three-dimensional free energy landscapes (FEL) illustrating conformational stability of the PPARG–mebendazole (**M**) and SLC30A10–NVP-TAE226 (**N**) complexes based on MD trajectories. Principal component analysis (PCA) covariance matrix heatmaps of the PPARG–mebendazole (**O**,**P**) and SLC30A10–NVP-TAE226 (**Q**,**R**) complexes, reflecting dynamic residue correlations.

The CMap database was employed to identify promising small-molecule drugs for UC treatment based on UC-related DEGs. The top 10 compounds with the highest potential for UC treatment are listed in Table 1. To validate their suitability, molecular docking analysis was performed between these ten drugs and the three model genes using AutoDock Vina, with results presented in Table 1. Mebendazole and NVP-TAE226 showed the lowest binding free energies (less than −8.0 kcal/mol) with PPARG and SLC30A10, respectively, indicating the most stable binding interactions. The drug binding poses and sites on the model genes are shown in Figure 7A,B. Mebendazole formed two hydrogen bonds with PPARG (Figure 7A), while NVP-TAE226 formed six hydrogen bonds with SLC30A10 (Figure 7B), stabilizing the drug–protein interaction.

To assess the stability of these complexes, 100 ns molecular dynamics simulations were carried out. Root Mean Square Deviation (RMSD) and root mean square fluctuation (RMSF) analyses (Figure 7C–F) indicated rapid equilibration and minimal fluctuations in residue positions, suggesting structural stability of both complexes. Solvent-accessible surface area (SASA) and radius of gyration (Rg) analyses (Figure 7G–J) further confirmed the compactness of the complexes’ conformations. The PPARG–mebendazole complex maintained 1–3 hydrogen bonds, while the SLC30A10–NVP-TAE226 complex consistently formed 4–6 hydrogen bonds (Figure 7K,L), suggesting a stronger interaction in the latter. Free energy landscape (FEL) analysis (Figure 7M,N) revealed that both complexes stabilized in dominant, low-energy conformational states, with the SLC30A10 complex showing slightly more flexibility due to its transmembrane nature. PCA and the corresponding FELs (Figure 7O–R) supported the presence of coordinated motions and stable conformational basins, further demonstrating the stability and strong binding affinity of both complexes. Overall, the PPARG–mebendazole and SLC30A10–NVP-TAE226 complexes exhibited strong binding interactions, structural stability, and minimal conformational drift, supporting their potential as promising therapeutic candidates for UC.

## 4. Discussion

UC, a chronic, relapsing inflammatory bowel disease, is characterized by intestinal inflammation, mucosal damage, and fibrosis [35]. Although its incidence continues to rise, the underlying molecular mechanisms remain unclear, and treatment outcomes are suboptimal. Sphingolipids, a diverse group of structurally and biologically active lipids, are metabolized through an intricate network of enzymes. Research into the role of bioactive sphingolipids in signaling mechanisms has expanded beyond their initial involvement in PKC regulation to encompass a range of biological processes, including metabolism, apoptosis, cellular development, differentiation, proliferation, immunology, inflammation, and related diseases [36]. This study leverages machine learning and bioinformatics approaches to identify SMGs as novel diagnostic biomarkers and potential therapeutic targets.

Through a series of analyses combining UC and SMGs, 29 common genes were identified. Functional enrichment analysis revealed that these genes are primarily involved in lipid localization, response to nutritional signals, and interleukin-4 and interleukin-13 signaling. These findings suggest that the identified genes may serve as key links between sphingolipid metabolism and UC, with the enriched pathways offering insight into the molecular mechanisms by which sphingolipid metabolism influences UC. Consensus clustering further classified patients with UC into two subgroups: C1 (a metabolically dysregulated subtype) and C2 (an immune-enriched subtype). This stratification reflects the clinical heterogeneity of UC, with some patients exhibiting severe inflammation and immune cell accumulation, while others display metabolic disorder characteristics. 

By integrating three machine learning methods, three model genes—CAV1, PPARG, and SLC30A10—were identified. The three-gene model demonstrated exceptional diagnostic performance, with an AUC of 0.991, highlighting its potential for non-invasive UC screening. The consistency of results across validation datasets and in vitro LPS-induced models further supports the reliability of these biomarkers. The identification of these genes as diagnostic biomarkers is consistent with their distinct roles in lipid metabolism and inflammation. CAV1, a membrane scaffolding protein found in lipid rafts and caveolae, plays a vital role in signal transduction, metabolism, endocytosis, and exocytosis [37,38,39]. By regulating the activation of mitogen-activated protein kinase family members, CAV1 suppresses pro-inflammatory cytokine production from macrophages and has been linked to the modulation of inflammation and innate immunity [39]. As the principal component of caveolae, structures rich in cholesterol and sphingolipids, CAV1 is involved in the dynamic regulation of cholesterol within cells. It regulates cholesterol distribution and transport on the cell membrane by binding to cholesterol [38]. In the present study, CAV1 was upregulated in UC and positively correlated with pro-inflammatory immune cells. Its overexpression may exacerbate mucosal inflammation by amplifying lipid-mediated signaling pathways, aligning with its involvement in lipid metabolic processes. In contrast, PPARG, a subfamily of PPARs involved in regulating immune tolerance, metabolism, and inflammation [40], and SLC30A10, a zinc transporter implicated in lipid synthesis [41], were both downregulated in UC. Moreover, the expression of PPARG and SLC30A10 was negatively correlated with anti-inflammatory cells, suggesting that their downregulation may promote inflammation by impairing the activity of anti-inflammatory cells and disrupting lipid homeostasis. These findings are consistent with prior studies linking PPAR deficiency to impaired mucosal repair [42] and SLC30A10 mutations to metabolic dysregulation [43].

Furthermore, shared TFs for these three genes were identified, with FOXA2 emerging as a key regulator potentially coordinating the expression of CAV1, PPARG, and SLC30A10. The FOX family of TFs plays a pivotal role in the differentiation and function of various cell types [44]. The expression of the gene regulating cystic fibrosis transmembrane conductance in intestinal epithelial cells depends on FOXA1/A2 [45,46]. FOXA2 also regulates the function of intestinal epithelial cells through a co-regulated gene network [47]. Deletion of FOXA1/A2 results in decreased intracellular adenosine 3′,5′-cyclic monophosphate levels, which are vital for ion and solute transport and other enterocyte processes [47]. These findings suggest that FOXA2 plays a pivotal role in intestinal epithelial differentiation and barrier integrity, and its dysregulation in UC could disrupt mucosal repair and lipid metabolism.

In this study, we identified mebendazole and NVP-TAE226 as promising therapeutic candidates for UC through integrative analysis using the CMap database and molecular docking. Molecular dynamics simulations further validated the stability and reliability of the predicted binding modes. Both the PPARG–mebendazole and SLC30A10–NVP-TAE226 complexes maintained stable interactions throughout the simulation, with low RMSD values, consistent hydrogen bonding, and favorable FELs, indicating strong and specific binding under physiological conditions. Mebendazole, an FDA-approved benzimidazole, is safe for use in both children [48] and adults [49] and is effective against various intestinal helminths. In addition to its antiparasitic properties, mebendazole has shown anti-inflammatory and anti-fibrotic effects in several cell lines and animal models by downregulating key signaling pathways such as mitogen-activated protein kinase [50,51], nuclear factor- kappa B [52], cyclooxygenase 2 [53], and TGF-β [23]. It also reduces collagen release and alpha-smooth muscle actin levels, contributing to its anti-fibrotic effects [54]. A recent animal study demonstrated that mebendazole reduces inflammation and accelerates healing in UC mouse models [55]. Furthermore, it was also reported to induce M2-phenotype polarization of macrophages with anti-inflammatory properties, resulting in the production of anti-inflammatory modulators, including IL-10 and CD206, and acceleration of the healing process [56]. In a pilot study, the addition of mebendazole to mesalamine for the treatment of UC was a safe and potentially beneficial approach to improve mesalamine efficacy and reduce clinical symptoms [57]. NVP-TAE226, a focal adhesion kinase (FAK) inhibitor, has been explored for treating various malignant tumors [58]. FAK serves as a key signaling node downstream of integrin and growth factor receptors, and its inhibition can block pro-inflammatory and pro-fibrotic signal transduction [59]. The high affinity of mebendazole for PPARG may restore its anti-inflammatory signaling, while the interaction between NVP-TAE226 and SLC30A10 could inhibit the activation of FAK by the zinc transporter, collectively contributing to the therapeutic effects in UC.

In summary, this study presents an innovative integration of sphingolipid metabolism and UC transcriptomic data to identify two distinct molecular subtypes of UC using consensus clustering. Three machine learning algorithms were employed in parallel for robust identification of key genes, with cross-validation among models effectively reducing the risk of overfitting. Validation using independent cohorts and experimental data further reinforces the credibility of the findings. Moreover, by combining CMap-based drug screening with molecular docking and molecular dynamics simulations, we performed a comprehensive evaluation of potential therapeutic compounds, offering new insights and directions for the clinical treatment of UC. However, several limitations remain in this study. First, the use of publicly available databases resulted in small sample sizes in the validation cohorts. Therefore, further validation through larger-scale, multicenter randomized controlled trials is needed. Additionally, functional experiments for verification are lacking. While LPS-induced Caco-2 cells validated the gene expression trends, further functional studies are essential to confirm the findings. Therefore, future studies using animal models such as DSS-induced colitis in mice are warranted to further validate the biological significance of the identified targets. 

## 5. Conclusions

Through the integration of SMGs and transcriptomics data, this study revealed two molecular subtypes of UC with significant biological functional differences and established a novel and excellent diagnostic model. Meanwhile, we identified mebendazole and NVP-TAE226 as potential candidates for the treatment of UC, providing a reference for the clinical management of UC.

## Figures and Tables

**Table 1:** Potential drugs analyzed by CMap and molecular docking.

Pert_Iname	Moa	Target_Name	Norm_Cs	Free Binding Energy (Kcal/Mol)		
				CAV1	PPARG	SLC30A10
vemurafenib	RAF inhibitor	BRAF|CYP2C19|CYP3A4|CYP3A5|RAF1	−1.9033	−7	−8.7	−8
NVP-TAE226	Protein tyrosine kinase inhibitor	IGF1R|PTK2	−1.7499	−6.2	−8.7	−9.1
PD-160170	Neuropeptide receptor antagonist	NPY1R	−1.7369	−5.9	−7.8	−7.5
U-0126	MEK inhibitor	MAP2K1|MAP2K2|JAK2|AKT1|CHEK1|GSK3B|LCK|MAP2K7|MAPK1|MAPK11|MAPK12|MAPK14|MAPK8|PRKCA|RAF1|ROCK1|RPS6KB1|SGK1	−1.733	−6.2	−7.3	−6.9
selumetinib	MEK inhibitor	MAP2K1|MAP2K2	−1.7202	−4.9	−7.1	−7.1
amperozide	Dopamine receptor antagonist	HTR2A|DRD2|FAAH	−1.7026	−7	−8	−7.4
PD-158780	EGFR inhibitor	EGFR	−1.6983	−5.4	−8.2	−6.8
UNC-0321	Histone lysine methyltransferase inhibitor	EHMT2	−1.6751	−5.4	−7.8	−7
mebendazole	Tubulin inhibitor	TUBA1A|TUBB|TUBB4B	−1.674	−6.8	−8.9	−8
PP-2	Src inhibitor	SRC|LCK|ABL1|LYN|RIPK2	−1.6735	−5.3	−8	−7.2

## Data Availability

The datasets generated and/or analyzed during the current study are available in the GEO (https://www.ncbi.nlm.nih.gov/geo/query/acc.cgi?acc=GSE48958, https://www.ncbi.nlm.nih.gov/geo/query/acc.cgi?acc=GSE75214, https://www.ncbi.nlm.nih.gov/geo/query/acc.cgi?acc=GSE38713, https://www.ncbi.nlm.nih.gov/geo/query/acc.cgi?acc=GSE87466 (accessed on 3 May 2025)).

## Supplementary Materials

The following supporting information can be downloaded at: https://www.mdpi.com/article/10.3390/cimb47080616/s1.
