# Supplementary material for: Molecular Subtypes and Biomarkers of Ulcerative Colitis Revealed by Sphingolipid Metabolism-Related Genes: Insights from Machine Learning and Molecular Dynamics
Source: Curr Issues Mol Biol. 2025 Aug 4;47(8):616. doi: 10.3390/cimb47080616 (PMC12384397; doi:10.3390/cimb47080616)
Supplement: Supplementary file 1 [file cimb-47-00616-s001.zip › Supplementary Figures.pdf]

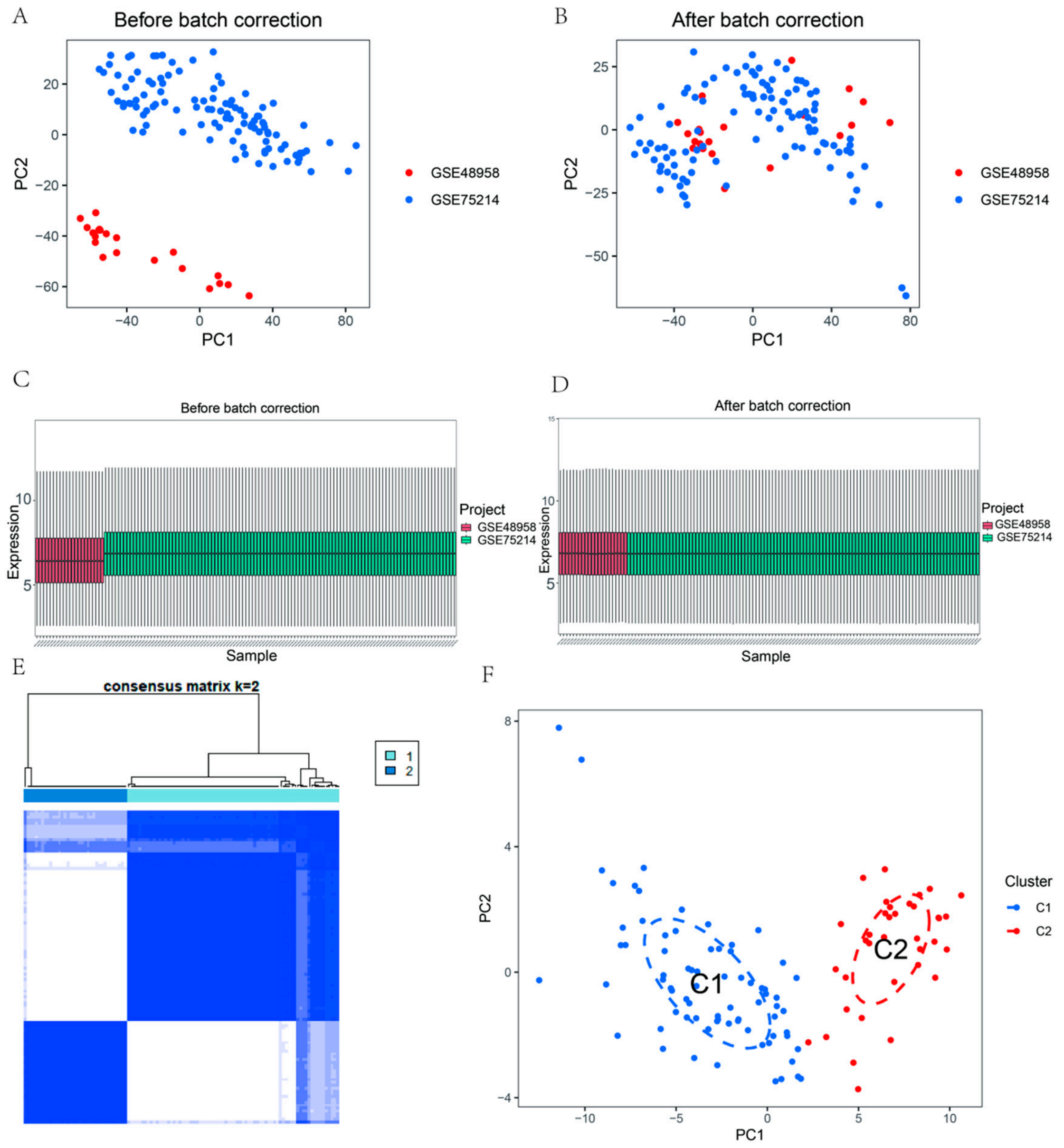

Fig. S1. Identifying SMG-related molecular clusters in UC. (A-D) PCA analysis and boxplots prior to and following the UC dataset merger; (E) Consensus clustering matrix with k=2. (F) PCA displays the distribution of subtypes.

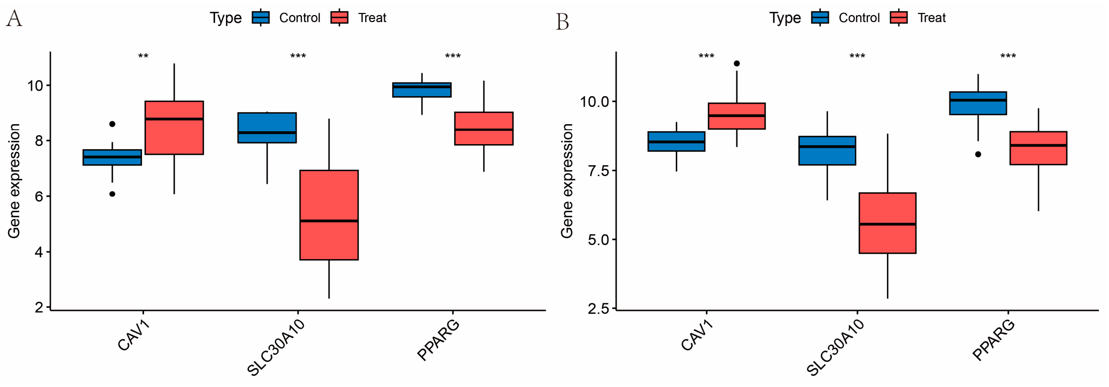

Fig. S2. The difference in CAV1, PPARG, and SLC30A10 expression between the normal and UC samples: (A) GSE38713; (B) GSE87466.

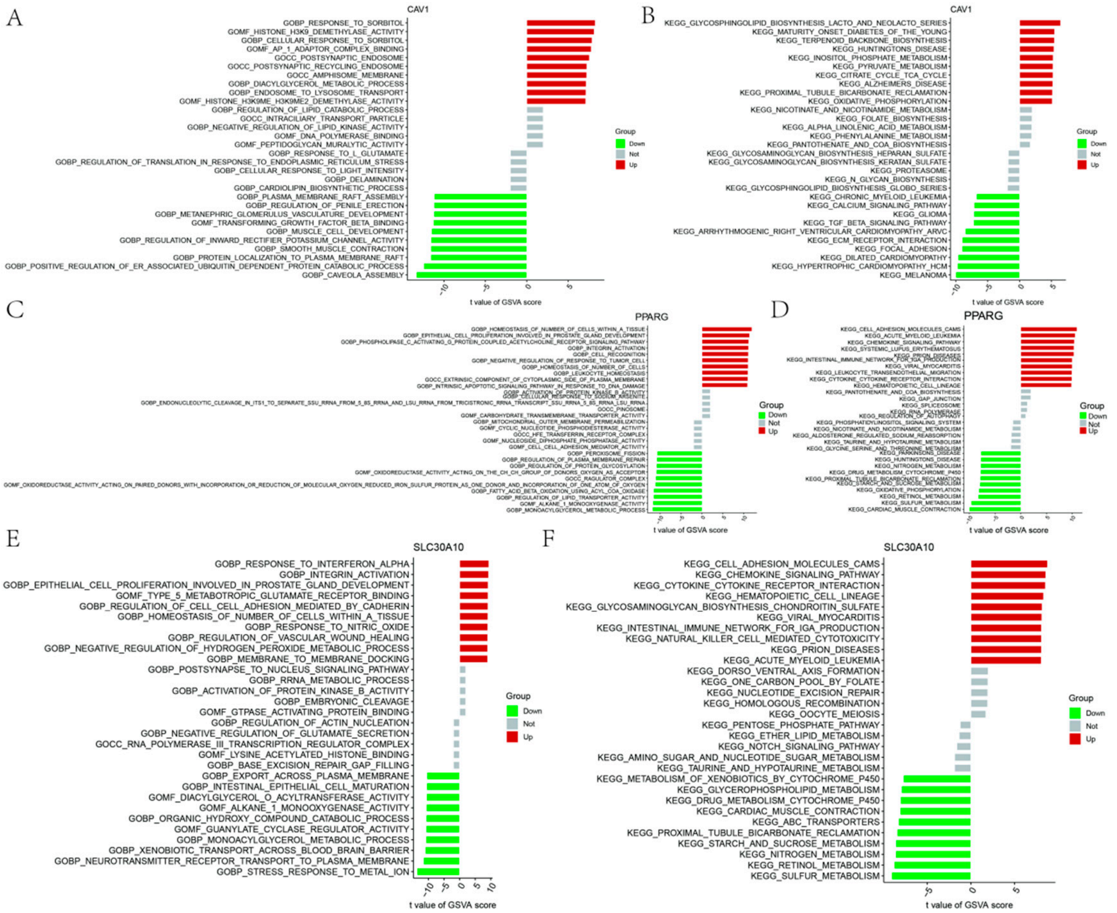

Fig. S3. GSVA of model genes. Comprehensive scoring of (A-B) CAV1, (C-D) PPARG and (E-F) SLC30A10 using the GSVA to explore their potential molecular mechanisms in UC.

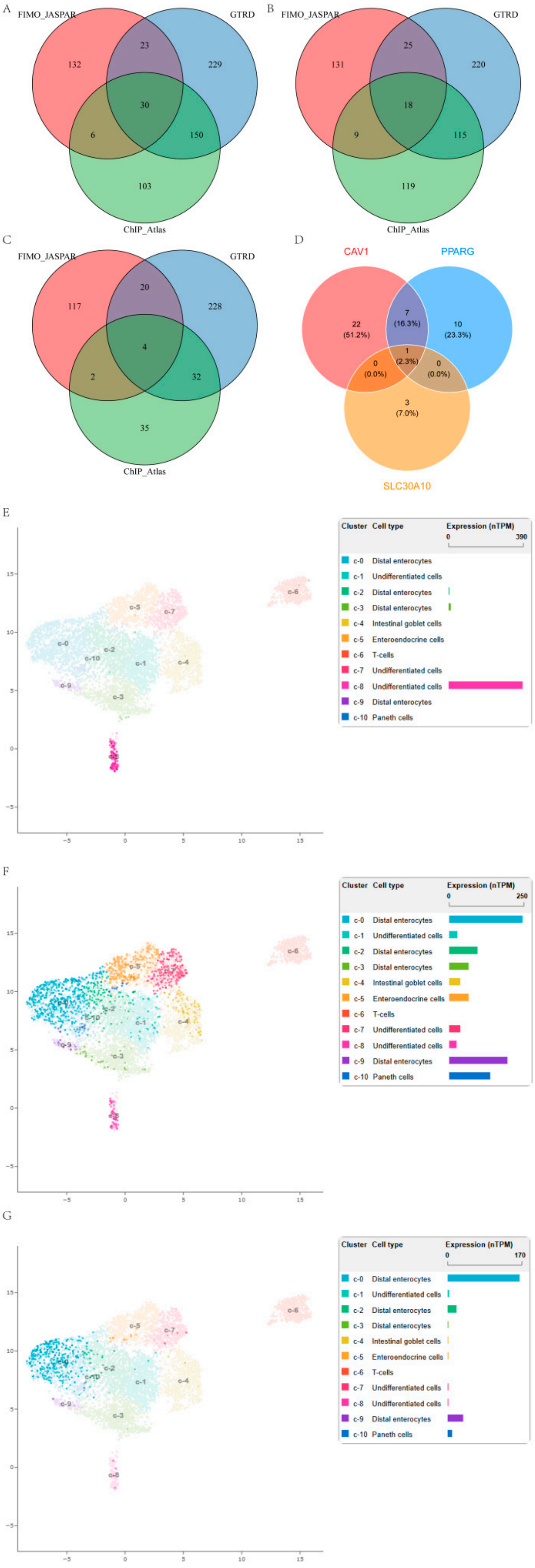
A
FIMO_JASPAR
GTRD
ChIP_Atlas
132
23
229
30
6
150
103
B
FIMO_JASPAR
GTRD
ChIP_Atlas
131
25
220
18
9
115
119
C
FIMO_JASPAR
GTRD
ChIP_Atlas
117
20
228
4
2
32
35
D
CAV1
PPARG
SLC30A10
22 (51.2%)
7 (16.3%)
10 (23.3%)
1 (2.3%)
0 (0.0%)
0 (0.0%)
3 (7.0%)
E
F
G
Cluster
Cell type
Expression (nTPM)
c-0 Distal enterocytes
c-1 Undifferentiated cells
c-2 Distal enterocytes
c-3 Distal enterocytes
c-4 Intestinal goblet cells
c-5 Enteroendocrine cells
c-6 T-cells
c-7 Undifferentiated cells
c-8 Undifferentiated cells
c-9 Distal enterocytes
c-10 Paneth cells

Fig. S4. TFs and single-cell mapping analysis of model genes. (A-C) TFs for CAV1, PPARG, and SLC30A10 predicted using various databases; (D) Intersection of TFs for the three model genes; (E-G) Single-cell mapping of CAV1, PPARG, and SLC30A10 using the HPA database.
