# Supplementary material for: Molecular Subtypes and Biomarkers of Ulcerative Colitis Revealed by Sphingolipid Metabolism-Related Genes: Insights from Machine Learning and Molecular Dynamics
Source: Curr Issues Mol Biol. 2025 Aug 4;47(8):616. doi: 10.3390/cimb47080616 (PMC12384397; doi:10.3390/cimb47080616)
Supplement: Supplementary file 1 [file cimb-47-00616-s001.zip › Supplementary Tables.pdf]

Supplementary Table 1. Information on the clinical characteristics of the samples used in the GEO dataset.

Dataset	UC samples	Normal samples
GSE48958	13 patients with UC (7 active and 6 inactive)	8 normal samples
GSE75214	97 patients with UC	11 normal samples
GSE38713	30 patients with UC (8 inactive UC, 7 uninvolved active UC and 15 involved active UC)	13 normal samples
GSE87466	87 patients with UC	21 normal samples
